# Upstream Drivers of Receiving Care in Minority-Serving Hospitals: a Cross-Sectional Study Using MCBS Data

**DOI:** 10.1093/geroni/igaf122.3680

**Published:** 2025-12-31

**Authors:** Louisa Holaday, Alina Kung, Yingtong Chen, Karen McKendrick, Bian Liu, Albert Siu

**Affiliations:** Icahn School of Medicine at Mount Sinai, New York, New York, United States; Icahn School of Medicine at Mount Sinai, New York, New York, United States; Icahn School of Medicine at Mount Sinai, new york, New York, United States; Icahn School of Medicine at Mount Sinai, New York, New York, United States; Icahn School of Medicine at Mount Sinai, New York, New York, United States; Icahn School of Medicine at Mount Sinai, new york, New York, United States

## Abstract

**Intro:**

Racial separation of care contributes to racial disparities in health. Little is known about upstream drivers of receiving care in a minority-serving hospital (MSH) and differences by race/ethnicity.

**Methods:**

Cross-sectional analysis of MCBS data (2010-2019) with binary outcome of hospitalization in MSH versus not. Exposures of interest were individual race/ethnicity and insurance; neighborhood disadvantage (SDI); and regional residential racial segregation. We included individual and regional covariates. In fully adjusted models, we tested whether race/ethnicity modified the association between SDI or residential segregation and hospitalization in MSH.

**Results:**

Among 8,735 hospitalizations, race/ethnicity (aOR 3.19 for Black vs. White patients; 95% Confidence Interval (CI): 2.60-3.91; P < 0.001); dual-eligible status (aOR: 1.30, 95% CI: 1.05-1.60; P < 0.05), neighborhood disadvantage (aOR for most disadvantaged versus least: 2.03, 95% CI: 1.56-2.63, P < 0.001); and residential segregation (aOR for high segregation versus low: 2.99, 95% CI: 1.98-4.52; P < 0.001) were independently associated with hospitalization in MSH. In the least disadvantaged or segregated areas, all patients were unlikely to be hospitalized in MSH; however, in the most disadvantaged or segregated areas, White patients had only a small increase in odds of MSH hospitalization while Black patients had a very large increase (P for interaction terms = 0.001 and 0.04, respectively).

**Conclusion:**

Our findings on differences by race/ethnicity suggest a need for further research on factors driving site of care, to address the upstream socio-structural determinants of health, and to invest in minority-serving hospitals given the practical challenges of eliminating neighborhood disadvantage and residential segregation.

